# Diversification dynamics, species sorting, and changes in the functional diversity of marine benthic gastropods during the Pliocene-Quaternary at temperate western South America

**DOI:** 10.1371/journal.pone.0187140

**Published:** 2017-10-26

**Authors:** Marcelo M. Rivadeneira, Sven N. Nielsen

**Affiliations:** 1 Laboratorio de Paleobiología, Centro de Estudios Avanzados en Zonas Áridas CEAZA), Av. Bernardo Ossandón, Coquimbo, Chile, Chile; 2 Departamento de Biología Marina, Universidad Católica del Norte, Larrondo, Coquimbo, Chile; 3 Instituto de Ciencias de la Tierra, Facultad de Ciencias, Universidad Austral de Chile, Isla Teja s/n, Casilla, Valdivia, Chile; University of California, UNITED STATES

## Abstract

Functional diversity based on species traits is a powerful tool to investigate how changes in species richness and composition affect ecosystem functioning. However, studies aimed at understanding changes in functional diversity over large temporal and spatial scales are still scant. Here we evaluate the combined effect of diversification and species sorting on functional diversity of fossil marine gastropods during the Pliocene-Quaternary transition in the Pacific coast of South America. We analyzed a total of 172 species in 29 Pliocene and 97 Quaternary sites. Each species was characterized according to six functional traits: body size, feeding type, mobility, attachment, life-habit, and larval mode. Functional diversity was estimated according to four indexes (functional richness, evenness, divergence and dispersion) based on functional traits measured. Extrapolated species richness showed a slight yet not significant decrease from the Pliocene to the Quaternary despite the fact that a large faunal turnover took place; furthermore, a large extinction of Pliocene species (61–76%) was followed by a high pulse of appearances (49–56%) during the Quaternary. Three out of four indices of functional diversity (evenness, divergence and dispersion) increased significantly towards the Quaternary which is more than expected under a random turnover of species. The increase in functional diversity is associated with a loss of large-sized carnivore forms, which tended to be replaced by small-sized grazers. Hence, this trait-selective species turnover, even in the absence of significant changes in species richness, likely had a large effect and has shaped the functional diversity of present-day assemblages.

## Introduction

The current threats to global biodiversity have increased the need to understand the role of species diversity on ecosystem functioning [1–4]. While the importance of species richness on ecosystem functioning has been demonstrated experimentally [3, 5, 6], its application across very large scales is impractical, limiting the generality of conclusions. A simple yet powerful approach to understand ecosystem functioning is through the concept of functional diversity (FD hereafter). FD measures “functional trait diversity, where functional traits are components of an organism’s phenotype that influence ecosystem level processes” [7]. A large body of literature has focused on theoretical or methodological aspects of FD analyses [7–11].

Unlike spatial patterns of variation in FD that have been increasingly studied in multiple taxa and systems [12–14], patterns of temporal variation are much less understood or have been documented over shorter timescales at small spatial scales [15]. The fossil record provides an excellent arena for the study of temporal variation in FD at much larger timescales and over larger spatial scales [15, 16]. Temporal changes in FD at evolutionary timescales can occur due to shifts in species richness (due to differential origination/extinction/dispersal rates) or species composition (due to species sorting). Species sorting (or species selection) refers to the preferential persistence of species according to the interaction between their intrinsic traits and the environment [17]. Therefore, non-random extinctions or originations may have important effects on FD, especially if the redundancy of the system is low [4, 8, 18]. Vast volumes of paleontological studies have documented the existence of species sorting in marine taxa [17, 19]. The selectivity of other traits seems sensitive to extinction intensity; for instance, a long list of different factors—including local abundance, reproductive mode, body size, trophic strategy, and life habit—were not selective at genus level during the K/Pg mass extinction [20, 21]. However, many of these factors have shown high selectivity during background extinction times or large extinctions at regional scales [22–24]. Whether the diversification trends and species sorting is translated into changes in FD remains much less studied [15, 16].

The Pliocene-Quaternary mollusc turnover provides an interesting temporal frame to evaluate these ideas. This faunal turnover, characterized by enhanced extinction and origination rates, affected different regions around the globe with variable intensity [22, 25–29] and was associated with major climatic and oceanographic generalized re-organization (e.g. cooling and changes in marine productivity) [30]. The impact of these transformations is well illustrated along the temperate coast of western South America where for more than a century a large Plio- Quaternary marine faunal turnover has been described [31] which altered the standing species richness and biogeographic patterns of several taxa [22, 32, 33]. If the faunal turnover was also selective for the ecological and life-history traits of these species, as shown in bivalves [22] and vertebrates [33], then an evolutionary change in the FD should be expected—a possibility that remains untested. Here we test these ideas, using benthic gastropods, a diverse and well-documented group with an excellent fossil record in the region [32, 34], as a study model. Our goals were: a) to quantify the magnitude of the Pliocene-Quaternary turnover in marine benthic gastropods along western South America, and b) to determine the impact of species sorting during the Pliocene-Quaternary turnover on the functional diversity of gastropod assemblages.

## Materials and methods

### Database

We gathered a database documenting the occurrence of 172 gastropod species from 126 fossiliferous sites (29 Pliocene, 97 Quaternary) along the temperate Pacific coast of South America from northern Peru to southern Chile. Although the paleoenvironments have been little studied, the environments represent shallow-water habitats (i.e., coastal shelf) in wave-exposed and wave-protected areas [34–36]. A total of 1,553 occurrences were compiled (see S1 Table). The information was collected from our own field studies and complemented with an exhaustive literature search [32, 37–58]. For each species, we gathered information about the body size (maximum shell length), locomotion (actively mobile, facultative mobile, slow-moving), attachment (attached, non-attached), life-habit (epifaunal, infaunal), feeding type (carnivore, grazer, suspension feeder), and larval mode (aplanktonic, planktonic feeding, planktonic non-feeding). Maximum shell length and larval mode were obtained from our own collections and observations and complemented with information from literature (see above and Supplemental information). Information for locomotion, attachment, life-habit and feeding type was gathered from Paleobiology Database (https://paleobiodb.org/), and its use has been validated by previous studies [59–61]. If information was not available for a given species, traits were inferred or generalized from species belonging to the same genus or family, given that ecological and life-history traits are often phylogenetically conserved [12, 62]. The relationship among these ecological and life history traits is not orthogonal (i.e. not all the combinations of these factors are observed/possible) because some traits are correlated in part. For instance, carnivores are typically mobile forms and tend to be large-sized. However, this possible multi-collinearity effect is properly addressed by functional diversity analyses (see below). The database of species occurrences and functional traits used in our analyses are provided in the supporting information (S1 and S2 Tables).

### Analyses

We evaluated the intensity of the Pliocene-Quaternary turnover, quantifying the standing species richness and the intensity of extinction and appearance of new forms. To evaluate the standing species richness in Pliocene and Quaternary assemblages we used the non-parametric extrapolation Chao 2 index [63] based on species-sites presence-absence matrices. We also estimated the species richness at a local scale using a subset of sites for which abundance (number of individuals per species) was available, using the Chao 1 index [64]. Analyses were carried out in the library vegan in R [65]. Species extinction was estimated as the percentage of Pliocene species not found during the Quaternary; conversely, species appearance was estimated as the percentage of Quaternary species making a first appearance during the Quaternary. These estimates, however, assume that sampling effort was similar between Pliocene and Quaternary, which clearly is not the case. In order to account for this possible bias, we re-estimated the Chao 2 index, extinction, and appearance using similar sampling effort for Pliocene and Quaternary assemblages (n = 29 sites in 10,000 runs).

Temporal changes in the prevalence of the six functional traits were evaluated using t-tests and χ^2^ tests. We estimated the importance of taxonomic selectivity for both extinction and apparition of taxa by comparing the observed proportion of families that went extinct and appeared with the expectations of a random null model which assumed that the faunal turnover was independent of the taxonomic identity of the species (10,000 runs).

All six functional traits were used to evaluate changes in the functional diversity and were characterized through four complementary metrics: a) functional richness (FRic), b) functional evenness (FEve), c) functional divergence (FDiv), and d) functional dispersion (FDis). These four metrics emphasize different aspects of the functional diversity of assemblages [15, 66]. FRic, FEve and FDiv are based on principal coordinates analyses which are based on a dissimilarity matrix built on Gower’s index [67]. FRic is measured as the convex hull volume of the multivariate trait space, where higher values indicate a larger functional space. FEve is measured as the minimum spanning tree linking all the species within the trait space, where high values of FEve imply a more regular filling of the functional space [67]. FDiv measures the species deviance from the centroid of the multivariate trait space, and it is higher if species are far from the centroid, implying the existence of more extreme trait values in the functional space [67]. FDis is the average distance to the centroid of the multivariate trait space, where higher values indicate a higher dispersion of species over the trait space [66]. Importantly, FD indices are not affected by the possible multi-collinearity among functional traits; indeed, the principal coordinate analysis transforms a number of correlated variables into a reduced subset of uncorrelated variables (PCO axes). We pooled species across all sites in Pliocene and Quaternary assemblages and estimated FD indices for each time bin. We estimated the change in FD according to each metric for: i) the Pliocene extinction (Pliocene FD—extinct species FD), ii) Quaternary appearance (appearing species FD- surviving species FD), and iii) between the Quaternary and Pliocene. We tested whether the change in each FD metric was different than expected under a null model of random species turnover by re-shuffling the species identity in the matrix of traits (10,000 runs). Analyses were carried out using the library FD [66] and scripts written in R [65].

## Results

**Fig 1:**
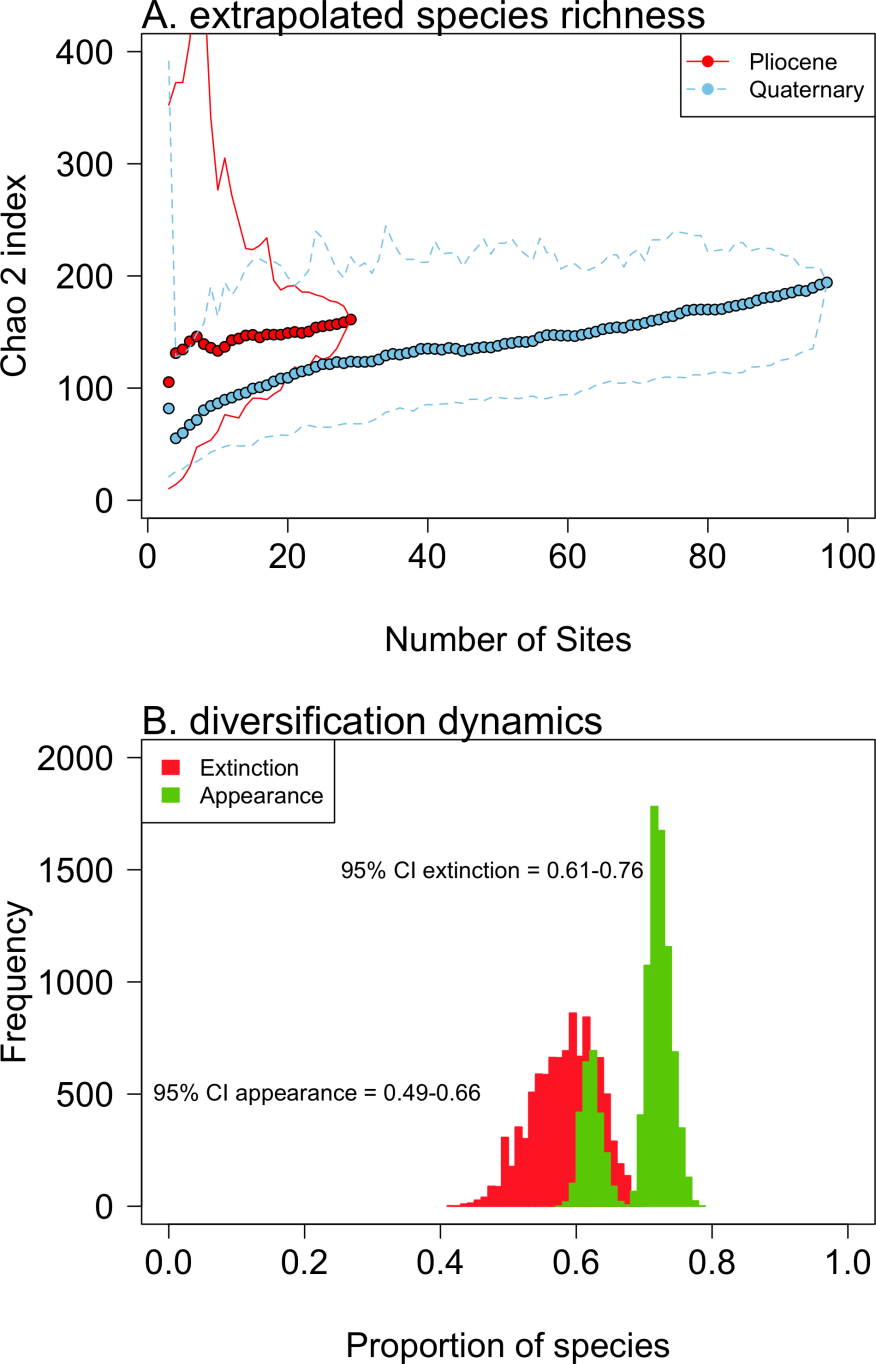
Diversification trends during the Pliocene-Quaternary faunal turnover. (A) Extrapolated species richness (Chao 2 index) versus sampling effort (sampled sites) for 29 Pliocene and 97 Quaternary sites. 95% CI are shown by lines. (B) Bias-corrected estimate of the Pliocene extinction and Quaternary apparition of species. Frequency distributions based on 10,000 resampled values with a similar sampling effort in Pliocene and Quaternary deposits (n = 29 sites).

The extrapolated species richness (Chao 2 index) increased with sampling effort (number of sites) in both Pliocene and Quaternary assemblages, showing no sign of saturation (Fig 1A). The observed Pliocene species richness (99) represents 61% (95% CI: 47–85%) of true “discoverable” richness. For Quaternary assemblages, the observed species richness (115) represents ca. 59% of the discoverable pool of species (95% CI: 42–97%). For a similar sampling effort, Pliocene assemblages showed a slightly higher extrapolated species richness, but this difference was not significant (Fig 1A). At a local scale, mean extrapolated richness (Chao 1 index) was not significantly different between Pliocene (median = 18 species, 7 sites) and Quaternary (median = 15 species, 31 sites) assemblages (t test, t = -1.11, P = 0.28). Face-value estimates show that 59% of species (95% CI: 47–67%; 57 out of 99 species) went extinct, and 63% (95% CI: 53–71%; 73 out of 117 species) originated during the Pliocene-Quaternary transition. When differences in sampling intensity were controlled, trends reversed (Fig 1B), showing higher extinction levels (95% CI: 61–76%) than appearance (95% CI: 49–66%).

**Fig 2:**
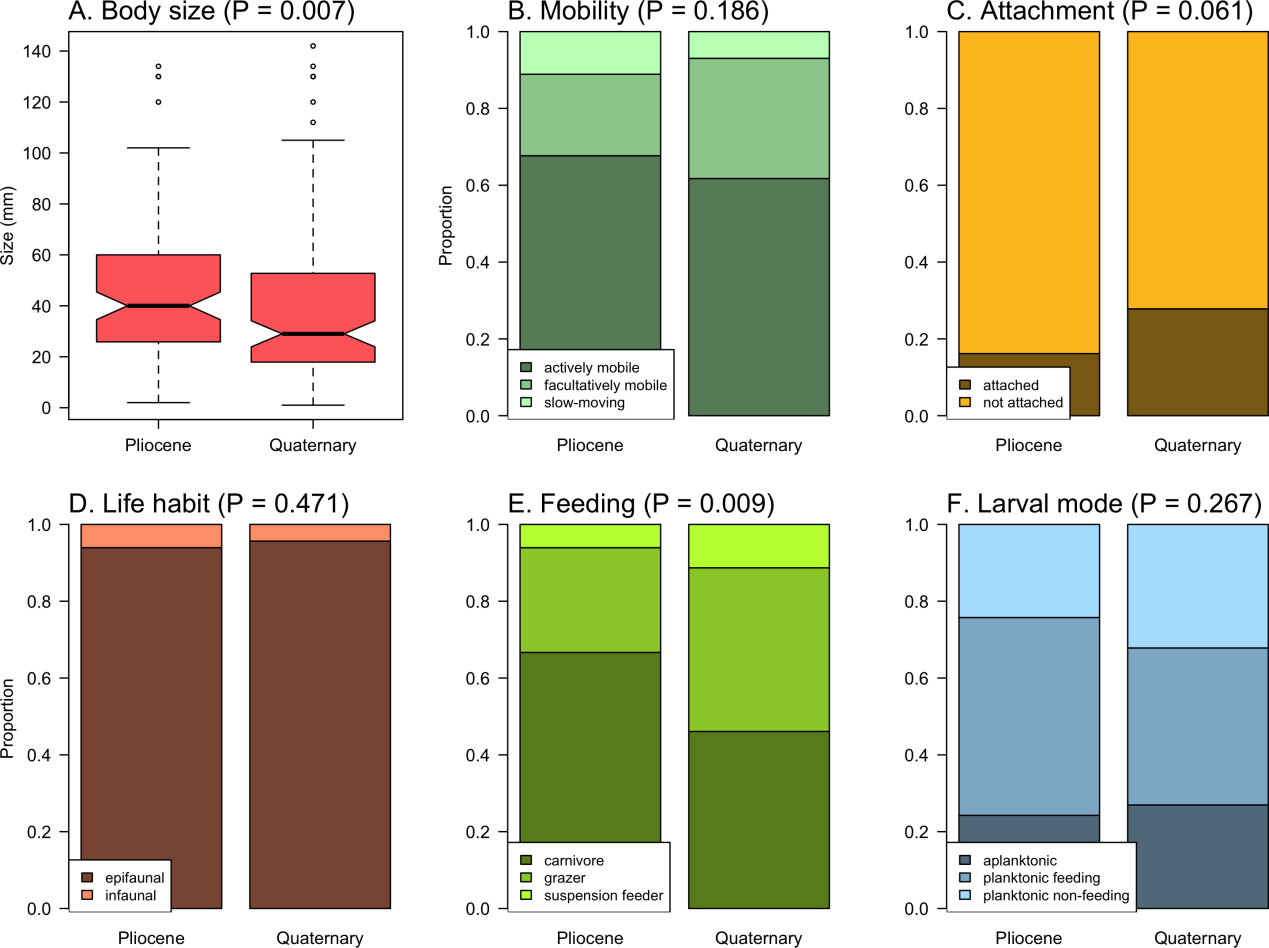
Temporal changes in the prevalence (i.e. proportion of species) of functional traits of gastropods. (A) body size, (B) mobility, (C) attachment, (D) life habit, (E) feeding, and (F) larval mode. The significance of changes is indicated by p-values (t-test for body size, χ^2^ test for other traits).

The prevalence of different ecological traits was variable in time (Fig 2). There was a significant reduction in the body size of species from the Pliocene (median = 40 mm) to the Quaternary (median = 29 mm) (t-test on log-transformed data, P = 0.007, Fig 2A). The prevalence of feeding types also experienced changes, particularly a reduction in the proportion of carnivores (from 65% to 46%) and an increase in the herbivore grazers (from 25% to 41%) (Fig 2E). For other traits, no significant changes were detected (χ^2^ test, P > 0.05, Fig 2).

**Fig 3:**
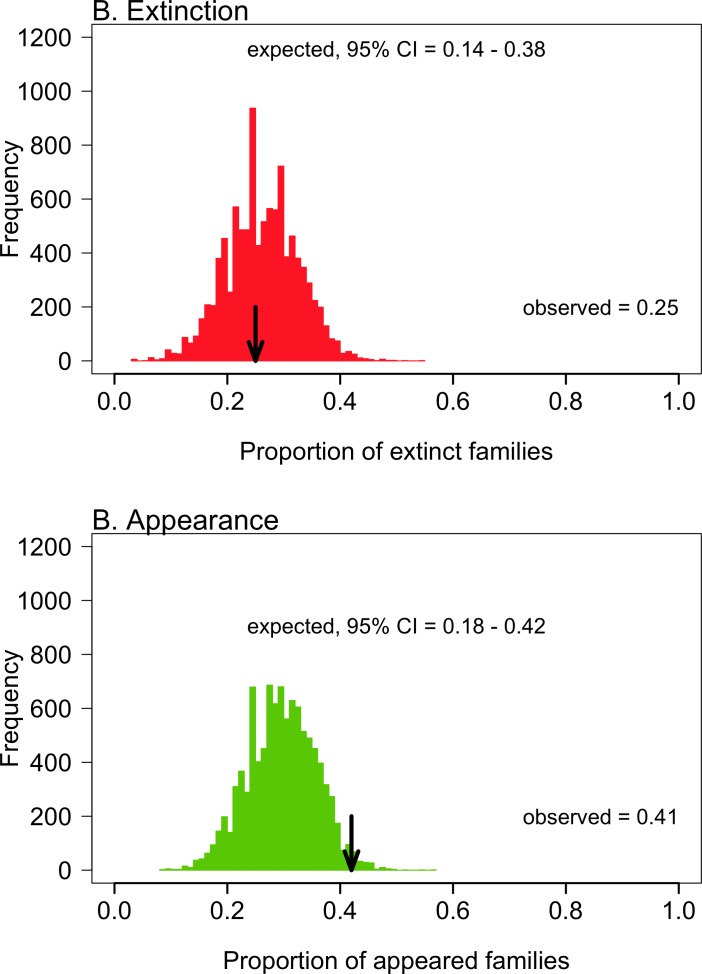
Taxonomic selectivity of Pliocene-Quaternary mollusc turnover. Observed proportion of extinct (A) and appearing families (B) versus the expected values under a null model assuming a taxonomically random species turnover (10,000 runs).

Neither the extinction nor the apparition of species was taxonomically selective, as the proportion of extinct/appearing families was not different than expected by the null model (Fig 3). Patterns were, in general, similar within the most speciose families (Table 1). For instance, in Muricidae—the most speciose family during both Pliocene and Quaternary—the observed proportion of extinct species (0.64, 23 out of 36) was not different than expected by chance (95% CI: 0.41–0.74). However, the proportion of appearing Muricidae during the Quaternary (0.38, 8 out of 21) was lower than expected by the null model (95% CI: 0.48–0.79).

**Table 1:** Proportion of extinct and appearing species within most speciose families across the Pliocene-Quaternary transition.

Family	Pliocene richness	Quaternary richness	Extinction*	Appearance*
Muricidae	36	21	0.64 (0.41–0.74)	**0.38 (0.48–0.79)**
Fissurellidae	9	12	0.44 (0.25–0.88)	0.58 (0.36–0.90)
Tegulidae	5	6	0.20 (0.00–1.00)	0.33 (0.20–1.00)
Naticidae	5	4	0.60 (0.00–1.00)	0.50 (0.20–1.00)
Nacellidae	5	3	1.00 (0.00–1.00)	1.00 (0.20–1.00)
Calyptraeidae	4	11	0.25 (0.20–1.00)	0.73 (0.29–1.00)
Buccinidae	4	5	0.50 (0.00–1.00)	0.60 (0.20–1.00)

*In parentheses are shown the expected values (2.5^th^ and 97.5^th^ quantiles) of a null model assuming no taxonomic structure (10,000 runs). Significant values are shown in bold.

**Fig 4:**
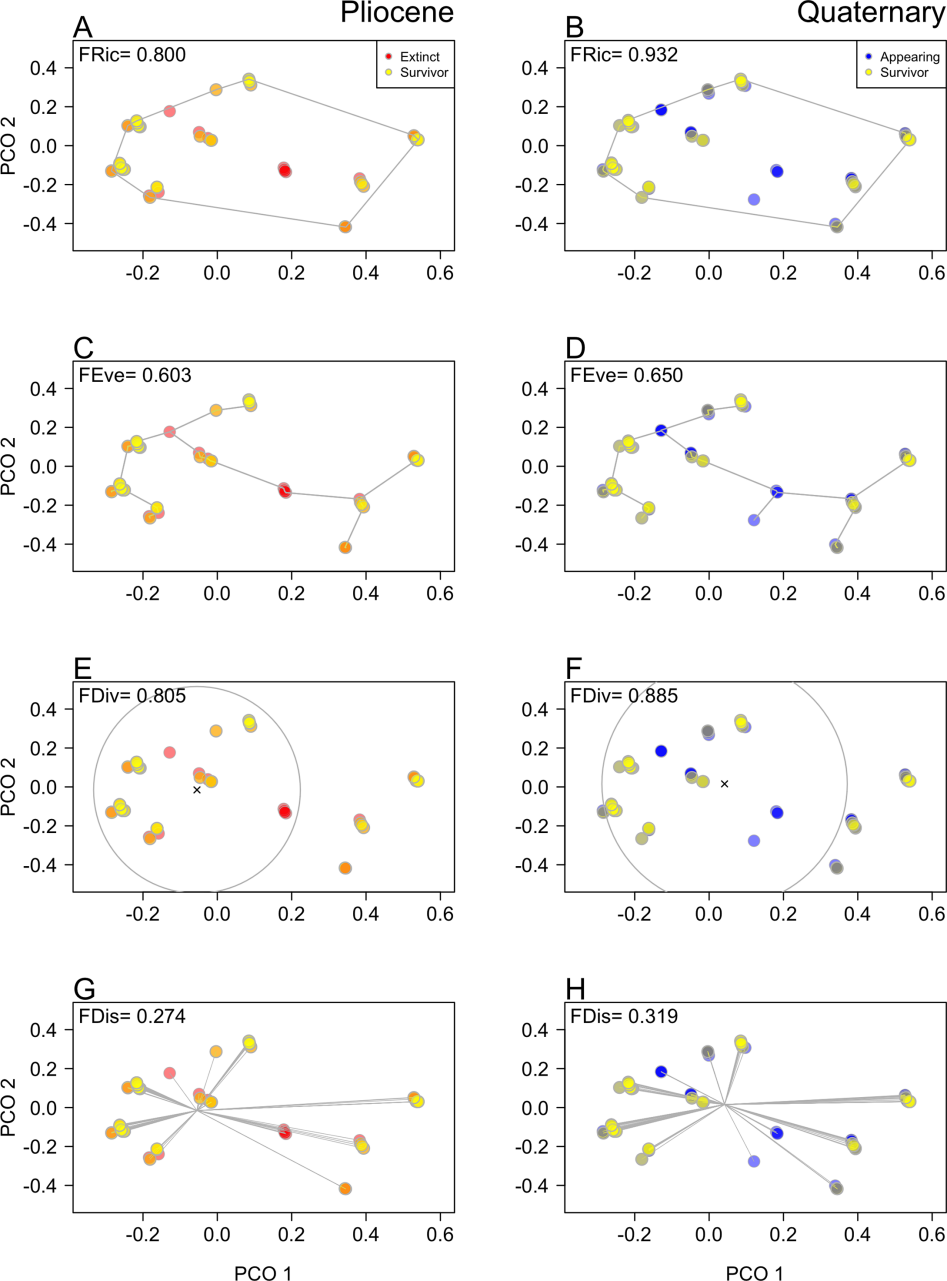
Changes in the functional diversity of gastropod assemblages during the Pliocene-Quaternary transition. (A-B) FRic: functional richness; (C-D) FEve: functional evenness; (E-F) FDiv: functional divergence; (G-H) FDis: functional dispersion). Each species is represented in a bivariate space created by the two first axes of a principal coordinate analysis (PCO) (see also Table 2).

The four metrics of functional diversity, depicted by the two first axes of a principal coordinate analysis, showed an increase from the Pliocene to the Quaternary (Fig 4), revealing changes in functional evenness, divergence and disparity, which was significantly higher than expected under a random null model of a trait-independent diversification (P < 0.05, Table 2). The Pliocene extinction produced a significant increase in all indices, except in the case of functional richness (Table 2). The Quaternary appearance of species also increased the functional divergence (P = 0.03), but the change for other indices were non-significant (Table 2).

**Table 2:** Changes in different functional diversity (FD) indexes across the Pliocene-Quaternary.

Comparison	FRic	FEve	FDiv	FDis
ΔFD after Pliocene extinction	0.25 (0.399)	**0.031** (0.011)	**0.041** (0.011)	**0.031** (0.045)
ΔFD after Quaternary appearance	0.326 (0.610)	-0.004 (0.340)	**0.071** (0.030)	0.010 (0.340)
ΔFD Quaternary-Pliocene	0.133 (0.203)	**0.047** (0.008)	**0.080** (<0.001)	**0.045** (0.004)

P-values (in parenthesis) based on 10,000 permutations. Significant values are shown in bold.

## Discussion

Our results show the FD of benthic gastropod assemblages along the western South America, indicating important changes during the Pliocene-Quaternary transition coupled with a large species turnover. Despite this species richness remaining relatively similar across time, the trait-selective replacement of a large number of taxa had a marked impact on the FD of gastropods.

The existence of a large faunal turnover during the Pliocene-Quaternary transition at western South America has been hypothesized for more than a century [31, 68]. Our results show the existence of a massive removal of species—confirming preliminary evaluations [51, 53, 56]—followed by the appearance of many new forms. These trends seem robust to differences in sampling intensity between the Pliocene and Quaternary and to the completeness of fossil inventories. Similarly, higher levels of biotic turnover have been estimated for bivalves and marine vertebrates [33]. The ultimate causes of the turnover are likely related to the onset of the modern conditions of the Humboldt Current and include several mutually non-exclusive factors, including a drastic decrease in sea temperature [69, 70], increasing primary productivity [71], destruction of wave-sheltered coastal habitats due to tectonic subsidence [34, 72], increasing anoxia due to increasing primary productivity [22], and habitat destruction and creation implied by the advance/retreat of glacial lobes in southern Chile during glacial/interglacial cycles [32]. Thus, the same factors promoting the disappearance of many forms also acted as drivers for the appearance of new species. The rapid appearance of new forms during the Quaternary has also been inferred based on phylogenetic analyses [73].

Evidence from the fossil record has shown drastic changes in FD through events of major biotic turnover [74], but patterns are hard to compare given the many methodological approaches used to assess patterns of functional diversity [75, 76]. Different FD trends can be inferred according to different methodological approaches [16, 77, 78]. Indeed, our analyses reveal that changes in FD can be inferred based on three out of four metrics. The functional richness index, sensitive to changes in species richness [67], increased slightly towards the Quaternary; however, the overall trait space occupied by species remains relatively similar, as revealed by the lack of significance in changes in functional richness. Indeed, no functional trait/group disappeared/appeared during the faunal replacement—a trend seen even during global mass extinction events [16]. However, the increase in functional evenness, divergence, and disparity indicates that the functional trait space is more homogenously filled and more dispersed towards the Quaternary. Berke et al. [12] have shown that functional evenness in marine bivalves increases towards higher latitudes which is attributed to an evolutionary accumulation of taxa due to biogeographic dynamics. The paleoenvironmental shift from subtropical to temperate conditions and the loss of many molluscs of tropical affinity during the late Neogene [79] are consistent with this pattern.

**Fig 5:**
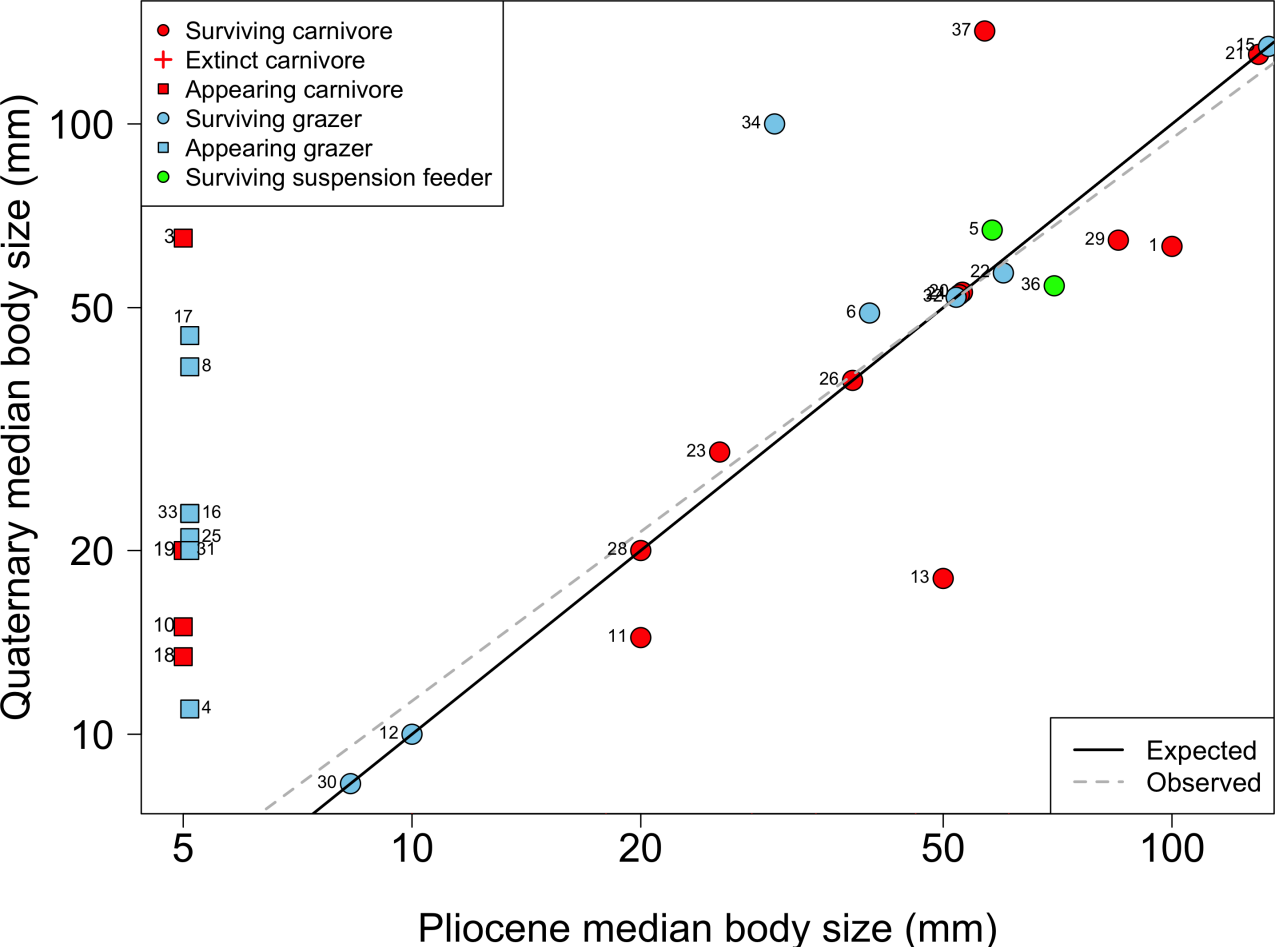
Changes in the median body size of 37 gastropod families across the Pliocene-Quaternary. Families were classified according to their feeding type and diversification dynamic. The solid line represents the null expectation of no change in median body size. The dotted line shows the fit of an OLS regression (r^2^ = 0.86, P < 0.001), where the slope (b = 1.15) was not different than the null expectation (P > 0.05). Also shown the median body size of families that went extinct or appeared. Families are depicted by numbers: 1 Buccinidae; 2 Bullidae; 3 Bursidae; 4 Calliostomatidae; 5 Calyptraeidae; 6 Cancellariidae; 7 Cassidae; 8 Cerithiidae; 9 Cliidae; 10 Columbellidae; 11 Drilliidae; 12 Ellobiidae; 13 Epitoniidae; 14 Fasciolariidae; 15 Fissurellidae; 16 Littorinidae; 17 Lottiidae; 18 Mangeliidae; 19 Marginellidae; 20 Mitridae; 21 Muricidae; 22 Nacellidae; 23 Nassariidae; 24 Naticidae; 25 Newtoniellidae; 26 Olividae; 27 Pseudolividae; 28 Pseudomelatomidae; 29 Ranellidae; 30 Rissoinidae; 31 Siphonariidae; 32 Tegulidae; 33 Trochidae; 34 Turbinidae; 35 Turridae; 36 Turritellidae; 37 Volutidae.

A strong species sorting during the faunal turnover was responsible for the increase in FD over time. Both extinction and appearance of forms were selective according to ecological and life-history traits, as commonly seen in the fossil record [17, 19], though not driven by changes in the taxonomic composition of assemblages. Changes in FD can be attributed to the preferential loss of large-sized carnivores and the subsequent increase in the proportion of small-sized herbivore grazers and suspension-feeders. On the one hand, the loss of carnivory could be explained by two non-exclusive processes: i) the elevated losses during the Pliocene extinction, and ii) the reduced appearance of new forms during the Quaternary. Five carnivore families went extinct during the Pliocene, compared to only three families appearing in the Quaternary (Fig 5). In parallel, the appearance of new species within Muricidae, the most speciose family analyzed, was lower than expected by chance. On the other hand, the increasing importance in herbivory could be attributed to a null extinction of families, the diversification of surviving forms (e.g. limpets and turbid snails), and to the appearance of seven new families (Fig 5). Interestingly, the overall reduction in median body size did not occur within surviving families; i.e. this was remarkably stable across the Pliocene-Quaternary transition, and it is rather attributed to the appearance of small-sized herbivore families (Fig 5). Indeed, in agreement with previous studies [56, 80] a few surviving families (e.g. Volutidae, Muricidae, and Fissurellidae] show an increase in median body size. However, this is not consistent with the so-called “Lilliput Effect”, i.e. a miniaturization of species body size immediately after a large extinction event [81, 82], because the decline in body size is largely due to newly originated species.

Previous studies have shown that the late Neogene extinction of bivalves and marine vertebrates along western South America were also trait-selective [22, 33]. This combined evidence suggests that the deep environmental transformations that took place during the Pliocene-Quaternary transition [71, 83] not only altered patterns of species diversity and composition but also might have brought a major shift in the functioning of marine ecosystems. Our results highlight the usefulness of FD indices to provide more detailed understanding of patterns of biotic turnover across evolutionary timescales.

## Supporting information

S1 Table Species occurrences in Pliocene and Quaternary sites. (XLS)

S2 Table Ecological and life history traits of gastropod species. (XLS)
