# Correction: Diversification dynamics, species sorting, and changes in the functional diversity of marine benthic gastropods during the Pliocene-Quaternary at temperate western South America

**DOI:** 10.1371/journal.pone.0191525

**Published:** 2018-01-16

**Authors:** Marcelo M. Rivadeneira, Sven N. Nielsen

In Fig 3, there is an error in the “Extinction” graph label. The graph label should be labeled “A. Extinction”. Please see the corrected Fig 3 here.

**Fig 3:**
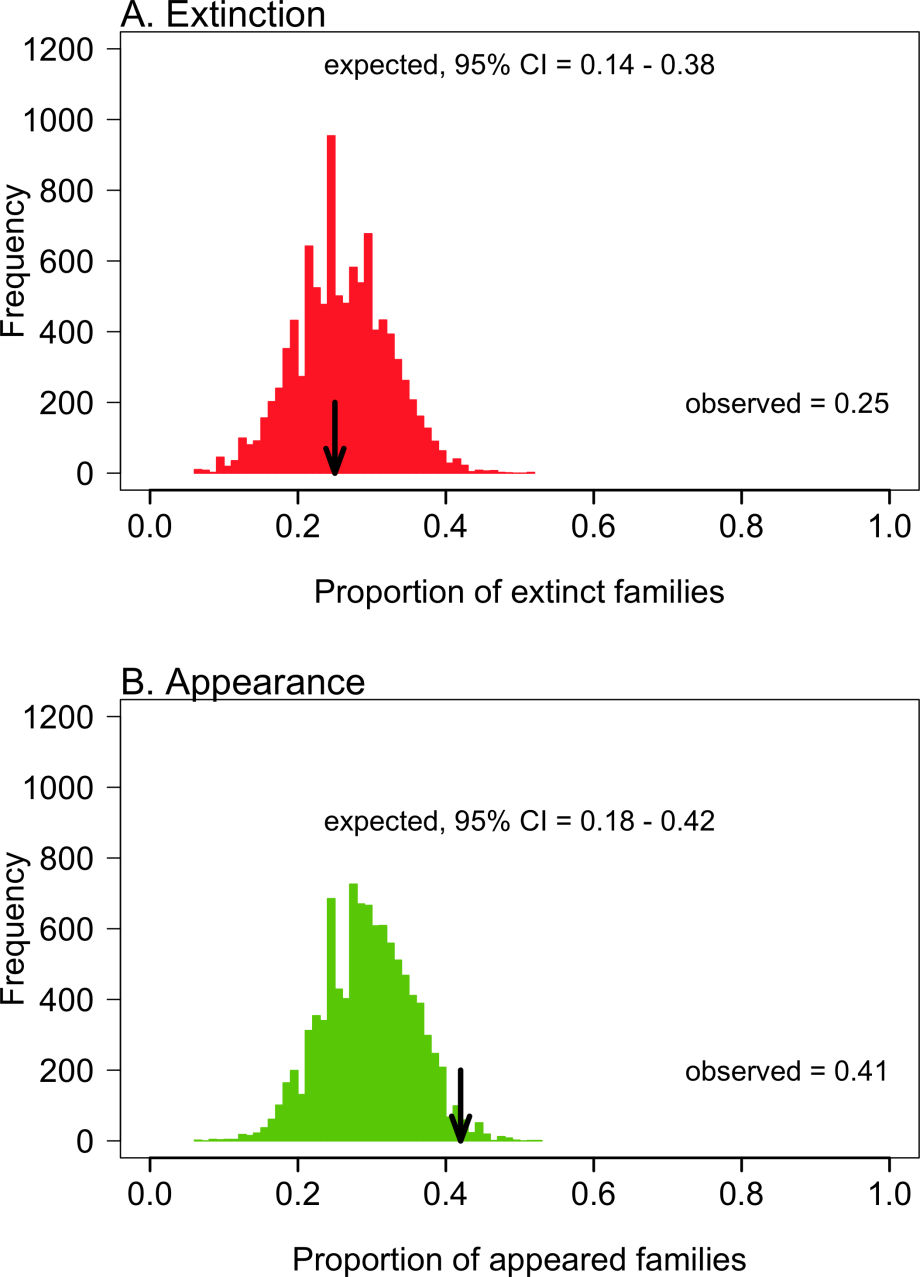
Taxonomic selectivity of Pliocene-Quaternary mollusc turnover. Observed proportion of extinct (A) and appearing families (B) versus the expected values under a null model assuming a taxonomically random species turnover (10,000 runs).

In Fig 5, there are missing and incorrect points. Please see the corrected Fig 5 here.

**Fig 5:**
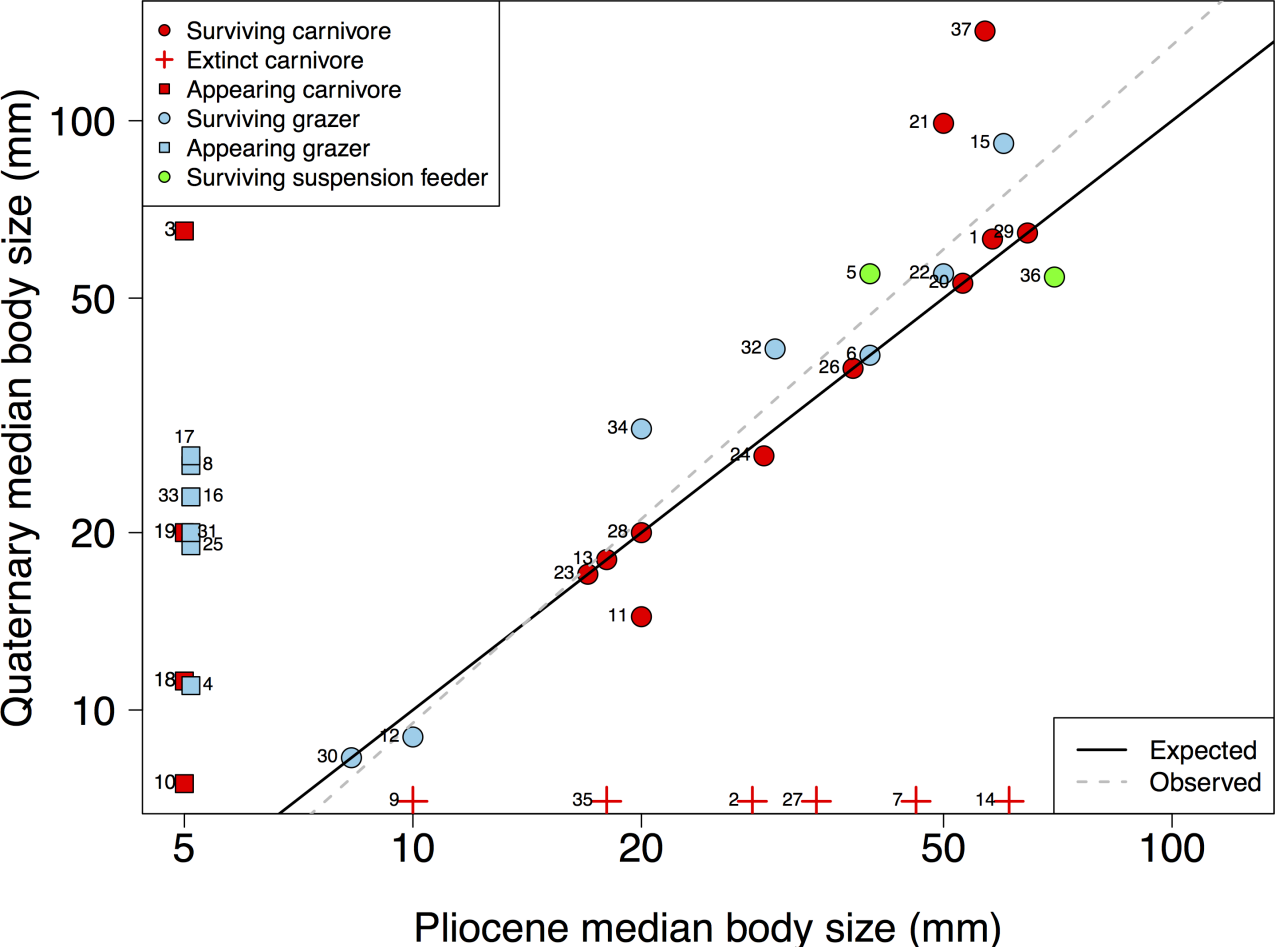
Changes in the median body size of 37 gastropod families across the Pliocene-Quaternary. Families were classified according to their feeding type and diversification dynamic. The solid line represents the null expectation of no change in median body size. The dotted line shows the fit of an OLS regression (r^2^ = 0.86, P < 0.001), where the slope (b = 1.15) was not different than the null expectation (P > 0.05). Also shown the median body size of families that went extinct or appeared. Families are depicted by numbers: 1 Buccinidae; 2 Bullidae; 3 Bursidae; 4 Calliostomatidae; 5 Calyptraeidae; 6 Cancellariidae; 7 Cassidae; 8 Cerithiidae; 9 Cliidae; 10 Columbellidae; 11 Drilliidae; 12 Ellobiidae; 13 Epitoniidae; 14 Fasciolariidae; 15 Fissurellidae; 16 Littorinidae; 17 Lottiidae; 18 Mangeliidae; 19 Marginellidae; 20 Mitridae; 21 Muricidae; 22 Nacellidae; 23 Nassariidae; 24 Naticidae; 25 Newtoniellidae; 26 Olividae; 27 Pseudolividae; 28 Pseudomelatomidae; 29 Ranellidae; 30 Rissoinidae; 31 Siphonariidae; 32 Tegulidae; 33 Trochidae; 34 Turbinidae; 35 Turridae; 36 Turritellidae; 37 Volutidae.
